# Community Mobility in Older Adults: Novel Methodologies, Risk Factors, and Interventions

**DOI:** 10.1093/geroni/igab046.2159

**Published:** 2021-12-17

**Authors:** Andrea Rosso, Michelle Carlson, Jana Hirsch

## Abstract

Community mobility is an individual’s movement outside the home. It is essential for the completion of many instrumental activities of daily living, such as shopping and healthcare, and promotes physical function, social engagement, independent living, and quality of life. Mobility research often focuses on gait speed measured in clinical settings, a critical but not sufficient determinant of community mobility. Here we present four talks that assess community mobility and its determinants using novel methodologies to enhance our understanding of how to maintain independence in older ages. First, Andrea Rosso presents characteristics of individuals with the strongest associations between environmental walkability, as assessed by virtual audits, and walking. Second, Kyle Moored demonstrates associations of self-reported fatigability with life space among older men, independent of their physical functioning. Breanna Crane introduces GPS-based objective measures of community mobility and their associations with cognitive and physical function of older adults. Finally, Pam Dunlap presents results of a randomized clinical trial of a physical therapy intervention to improve walking in older adults on subjective and objective measures of life space. These talks will provide a better understanding of the factors related to community mobility, introduce attendees to novel methodologies in the assessment of both community mobility and risk factors associated with the loss of community mobility, and demonstrate approaches to improve community mobility in at-risk older adults. The discussant, Jana Hirsch, will provide perspectives on how these data inform our current view of community mobility and will lead a discussion with the audience.

